# Semantic Accessibility Is Associated with Reduced Experience-Induced Heuristic Fixation in Creative Problem Solving

**DOI:** 10.3390/jintelligence14040068

**Published:** 2026-04-17

**Authors:** Shangqing Yuan, Yifei Fang, Luming Zheng, Jun Zhang, Hengrui Zhang, Tie Sun

**Affiliations:** 1 College of Education, Huainan Normal University, Huainan 232063, China; 18739966948@163.com; 2 Joint Education Institute of Zhejiang Normal University and University of Kansas, Zhejiang Normal University, Jinhua 321017, China; elizabeth@zjnu.edu.cn; 3 Faculty of Social Sciences, Leiden University, 2311 EZ Leiden, The Netherlands; l.zheng@fsw.leidenuniv.nl; 4 College of Home Economics, Hebei Normal University, Shijiazhuang 050025, China; zhangjun19940114@126.com; 5 School of Economics and Finance, Huaqiao University, Quanzhou 362021, China; henry@hqu.edu.cn

**Keywords:** creative problem solving, heuristic-analytic processing, semantic accessibility, situational induction, semantic richness

## Abstract

Creative problem solving often fails because people rely on heuristic responses reinforced by prior experience. According to the default–interventionist account, analytic intervention can override these heuristic defaults only when the semantic system provides access to competing representations. We tested this prediction using a modified Chinese Remote Associates Task in which two factors were independently manipulated: semantic accessibility (high vs. low) and situational induction (strong vs. weak). A significant interaction emerged: strong induction impaired performance only under low semantic accessibility, whereas high semantic accessibility was associated with attenuated induction costs. This pattern is consistent with semantic accessibility serving as a cognitive buffer that may support analytic override of induced heuristic defaults. A separate comparison between induction and non-induction trials confirmed that induction reliably produced a mental set. These findings resolve conflicting claims about the role of semantic knowledge in creativity by showing that knowledge both constrains and enables insight depending on its interaction with experience-driven heuristics.

## 1. Introduction

Creative problem solving is often described as a competition between rapidly generated heuristic responses and slower, effortful analytic processes. According to the default–interventionist model (Evans, 2006; Stanovich et al., 2011), people typically begin by constructing the most plausible interpretation or response based on task features, goals, and—critically—prior experience. These heuristic defaults are efficient, but they can block access to better solutions unless the analytic system intervenes to detect conflict and revise the initial model.

This framework provides a natural account of why experience-induced mental sets—such as repeatedly applying the same strategy—produce robust fixation effects. In classic Einstellung paradigms, a familiar solution pattern becomes the dominant heuristic response, suppressing conflict detection and delaying analytic engagement (Bilalić et al., 2008; Luchins, 1942). Neurocognitive evidence shows that repeated strategy use can dampen dorsal anterior cingulate cortex (dACC) activity during early processing (Huang et al., 2019), suggesting that strong heuristic activation makes analytic intervention less likely to occur.

Beyond its role in fixation, however, the same default–interventionist model also highlights a theoretically important—but unresolved—possibility: that background semantic knowledge may influence the likelihood of analytic intervention. Semantic richness has been found to promote broader associative activation, facilitate flexible semantic retrieval, and enhance conflict detection (Kenett, 2018). In the default–interventionist framework, these functions directly map onto the factors that determine whether the analytic system overrides the heuristic default: a richer semantic landscape may allow problem solvers to (1) activate alternative representations more readily, and (2) detect mismatches between the default response and task demands. A key operational expression of semantic richness is semantic accessibility—the degree to which diverse semantic pathways are available during search—which directly determines how readily competing representations can be activated.

This dual role of semantic knowledge yields a sharp theoretical tension. On one hand, semantic knowledge is known to reinforce dominant associations, increasing the probability that an obvious but misleading heuristic response will be produced—thereby strengthening fixation, especially in insight-like tasks (Wiley, 1998). On the other hand, semantic richness could facilitate analytic intervention by providing additional cues for conflict detection and representational restructuring. Thus, existing theories make opposing predictions about whether semantic accessibility exacerbates or buffers against experience-induced heuristic fixation. Surprisingly, no study has directly tested this interaction.

To resolve this tension, the present study addresses this gap using a modified Chinese Remote Associates Task (CRAT) that independently manipulated (a) semantic accessibility, which reflects the breadth of available pathways in semantic search (high vs. low), and (b) situational induction, which reinforces a specific strategy and therefore generates a strong or weak heuristic default response. From a default–interventionist perspective, strong induction should strengthen the heuristic model constructed at the start of each trial, making analytic intervention less likely. Conversely, high semantic accessibility reflecting a broader semantic network—should increase the availability of competing representations, enhancing the probability of conflict detection and analytic override.

This leads to a key prediction: semantic accessibility should selectively buffer against the negative effects of strong situational induction. When semantic accessibility is low, the heuristic response should dominate, and strong induction should yield maximal fixation. When semantic accessibility is high, analytic intervention should remain viable even under strong induction, reducing fixation. By testing these theoretically incompatible predictions, our study provides an explicit link between semantic memory structure and experience-driven heuristic dominance, offering a mechanistic account of how knowledge can both hinder and enable creative cognition.

## 2. Methods

### 2.1. Participants

A priori power analysis was conducted using G*Power 3.1 (Faul et al., 2007) for a 2 × 2 within-subjects ANOVA design. Following conventions recommended by Lakens (2013), we targeted a medium-sized interaction effect (*η_p_*^2^ = 0.06), which is considered a reasonable estimate for cognitive experimental studies involving factorial manipulations. Given an α level of 0.05 and desired power of 0.80, the estimated required sample size was 29 participants. We recruited 30 participants (24 females, *Mage* = 21.2 years, *SD* = 1.9) to meet this threshold. All participants were native Chinese speakers with normal or corrected-to-normal vision and reported no history of neurological or psychiatric conditions. Written informed consent was obtained prior to participation.

### 2.2. Materials

Stimuli were adapted from the Chinese Remote Associates Test (CRAT) following Wu et al. (2017) and Sun et al. (2023). On each trial, three cue characters were vertically presented (58-point Songti, white font, black background), and participants generated a single character that formed a meaningful left-headed compound with all three cues. For example, the target character “从” (meaning “from” or “follow”) can combine with the cue characters “头” (head), “军” (army), and “此” (this) to form the compounds “从头” (from the beginning), “从军” (join the army), and “从此” (from then on). Restricting solutions to the left side ensured uniformity in the spatial structure of cue–solution combinations, avoiding variability known to affect induction effects. Character complexity (stroke count 2–15) and structural properties were matched across conditions.

Semantic accessibility was operationalized as the breadth of available pathways through which a target character can be reached during semantic search. In the Chinese morphographic system, a single character can combine productively with a large number of other characters to form two-character compound words. A target character that forms many high-frequency compounds with diverse characters therefore has a broader semantic network and more available activation pathways—reflecting high semantic accessibility. Conversely, a target character connected to fewer characters at high frequency yields a narrower semantic search space—reflecting low semantic accessibility. This operationalization captures the breadth of semantic connectivity rather than the strength of any single pairwise association, and should therefore be distinguished from pairwise associative strength as typically measured in English RAT paradigms. It should be acknowledged, however, that associative frequency also correlates with overall item difficulty in the present design, such that high-accessibility items were somewhat easier overall. The present findings should therefore be interpreted as reflecting an interaction involving semantic accessibility and associative availability, rather than as definitively isolating the contribution of semantic network breadth per se.

Accordingly, associative frequency—extracted from the Dictionary of Frequently Used Words in Modern Chinese—was used as an operational index of semantic accessibility. High-accessibility items contained strong cue-solution associative frequency, providing more accessible pathways and a broader semantic search space. Low-accessibility items contained weak associative frequency, providing fewer accessible pathways and a narrower semantic search space. A paired-samples *t*-test confirmed that high-accessibility items exhibited significantly higher cue-solution associative frequency than low-accessibility items, *t* (29) = 3.63, *p* < .001, *Cohen’s d* = 0.32.

Situational induction was manipulated by varying the extent to which a specific solution pattern was reinforced prior to each test trial. Each induction phase began with an initial trial in which all cues shared the same target character, establishing an initial strategy. Subsequent enhanced-induction trials partially replaced the cue set while preserving the identical target, thereby repeatedly activating the same solution schema. In the weak-induction condition, only one enhanced-induction trial followed the initial induction trial. In the strong-induction condition, the same enhanced pattern was repeated three times, creating a longer reinforcement sequence and stronger activation of the heuristic response pattern. This repetition structure follows prior work demonstrating that three or more repeated activations of the same cue-solution schema are sufficient to produce a stable mental set at both behavioral and neural levels (Huang et al., 2019; Luchins, 1942). The rationale for this repetition structure and its empirical validation are discussed in further detail in Section 2.4.

### 2.3. Design

The study employed a 2 (Semantic Accessibility: high vs. low) × 2 (Induction Strength: strong vs. weak) within-subjects design. Each participant completed all four conditions (HA [High Accessibility, Strong Induction], HW [High Accessibility, Weak Induction], LA [Low Accessibility, Strong Induction], LW [Low Accessibility, Weak Induction]), each containing six test trials, totaling 24 test trials. To eliminate item-specific confounds, we used two counterbalanced versions of the stimulus set: every item served as a test item for half of the participants and as a control item (no induction) for the other half. Thus, differences between test and control trials could be attributed solely to induction, not to item characteristics or baseline difficulty. Trial order was pseudo-randomized and counterbalanced across participants. Accuracy was aggregated at the participant level for each condition prior to statistical analysis. The use of repeated-measures ANOVA on aggregated accuracy is consistent with standard practice in cognitive experimental research (Judd et al., 2012), and the fully counterbalanced within-subjects design ensures that item-level difficulty differences are balanced across conditions by design, mitigating the primary concern motivating trial-level modeling approaches.

### 2.4. Procedure

**Figure 1:**
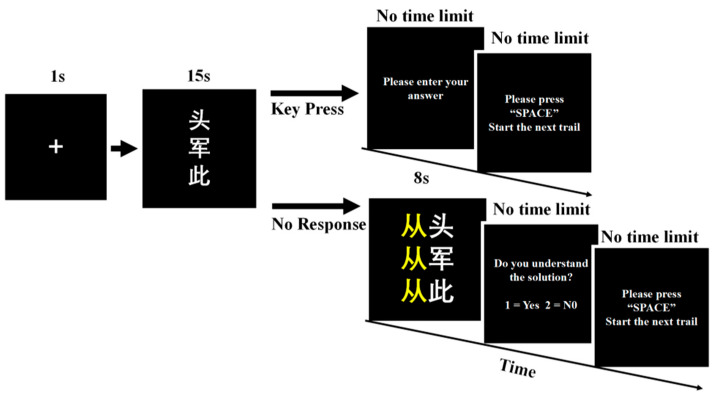
Procedure of a trial of the CRAT. The cue characters shown in the example are “头” (head), “军” (army), and “此” (this), and the target character is “从” (meaning “from” or “follow”), which forms the compounds “从头” (“from the beginning”), “从军” (“join the army”), and “从此” (“from then on”).

Figure 1 illustrates the procedure of a single CRAT trial. Participants first completed a practice session to familiarize themselves with the response procedure. Each trial began with a 1000 ms fixation cross, followed by presentation of the three cue characters. The cues remained onscreen until participants entered a solution or until 15 s elapsed. Participants pressed the “1” key to open a text box, within which they could revise their response. If no response was given within the time limit, the correct answer was shown for feedback. Participants were seated approximately 60 cm from the monitor.

Each induction-test sequence consisted of an initial induction trial, one or three enhanced induction trials (depending on condition), and a test trial. In the test trial, two cues were repeated from the final induction trial and one was novel. Crucially, the target character that solved the induction trials was never the correct solution for the test trial, requiring participants to override the reinforced response tendency. To prevent participants from inferring the task structure, a control trial-identical in format—but without any induction phase—appeared after each induction test sequence. As described above, counterbalancing ensured that each trial appeared in both test and control roles across participants. Responses containing characters that participants did not recognize were excluded from analysis.

The induction manipulation was designed to operationalize experience-driven heuristic dominance, a critical construct in heuristic-analytic theories of reasoning (Evans, 2006; Stanovich et al., 2011). These heuristic-analytic frameworks posit that repeated activation of the same solution pattern strengthens a default heuristic model, making it more likely to be used automatically and less likely to be overridden by analytic processes unless conflict is detected. Our induction sequences directly instantiate this mechanism by repeatedly reinforcing the same cue-solution schema. Prior work using similar repetition-based induction structures has demonstrated robust mental-set formation, reduced flexibility, and attenuated conflict-monitoring signals in the anterior cingulate cortex (Huang et al., 2019), confirming that this approach produces stable heuristic dominance at both behavioral and neural levels.

Increasing the number of enhanced-induction trials therefore provides a theoretically principled and empirically validated method for modulating the strength of the heuristic default. Weak induction yields minimal reinforcement, whereas strong induction extends the temporal persistence and activation strength of the same strategy, increasing the likelihood that participants rely on it in the subsequent test trial. The substantial performance decrement observed in test trials relative to control trials (see Section 3) confirms that the induction manipulation effectively induced a mental set and that its strength varied systematically across conditions. This provides both a conceptual and empirical foundation for using the induction strength factor to examine how semantic accessibility modulates the likelihood of analytic intervention. Example stimulus sets for the four experimental conditions are shown in Table 1.

## 3. Results

**Figure 2:**
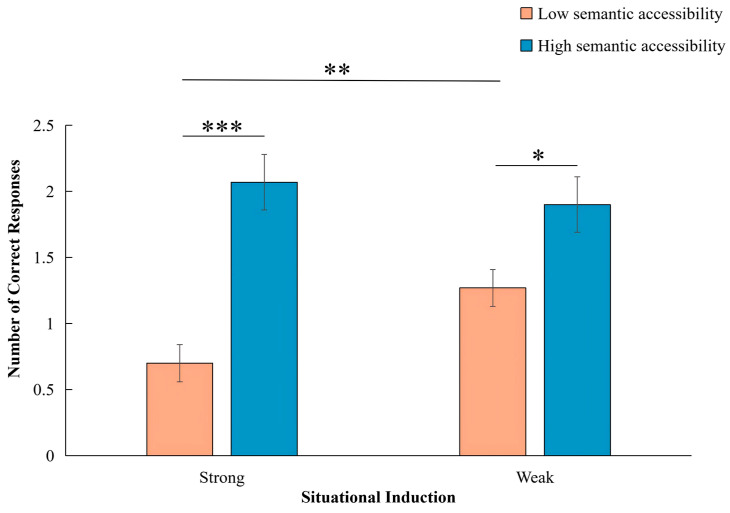
Mean number of correct responses across conditions varying in semantic accessibility (high vs. low) and situational induction (strong vs. weak). Note. Error bars represent standard errors of the mean. * *p* < .05, ** *p* < .01, *** *p* < .001.

The present study conducted a 2 (Semantic Accessibility: High vs. Low) × 2 (Situational Induction: Strong vs. Weak) repeated-measures ANOVA on the number of correct responses as shown in Figure 2. The main effect of Semantic Accessibility was significant [*F* (1, 29) = 24.86, *p* < .001, *η_p_*^2^ = 0.46], indicating higher accuracy under high semantic accessibility conditions (*M* = 1.98, *SE* = 0.16, 95% *CI* [1.65, 2.31]) compared to low semantic accessibility conditions (*M* = 0.98, *SE* = 0.10, 95% *CI* [0.77, 1.19]). The main effect of Situational Induction was not significant [*F* (1, 29) = 1.40, *p* = .250]. Importantly, a significant interaction emerged between semantic accessibility and situational induction [*F* (1, 29) = 4.88, *p* = .035, *η_p_*^2^ = 0.14], consistent with our prediction that semantic accessibility would attenuate the negative impact of induction.

Simple effects analysis, conducted using pairwise comparisons with Bonferroni correction, revealed that under low semantic accessibility, participants performed better in the weak induction condition (*M* = 1.27, *SE* = 0.14) than in the strong induction condition (*M* = 0.70, *SE* = 0.14) (*p* = .009, *d* = 0.52, 95% *CI* [0.154, 0.979]). However, under high semantic accessibility, no significant difference was observed between strong (*M* = 2.07, *SE* = 0.21) and weak induction (*M* = 1.90, *SE* = 0.21), *p* = .538 (*d* = 0.12, 95% *CI* [−0.38, 0.71]) (see Figure 2). At the same time, under both weak induction (*p* = .016) and strong induction conditions (*p* < .001), the number of correct answers for the high semantic accessibility condition was significantly higher than that for the low semantic accessibility condition. Thus, strong induction impaired performance only when semantic accessibility was low.

To directly address whether the observed interaction reflected a genuine attenuation effect rather than baseline difficulty differences between accessibility conditions, we computed an induction cost index for each participant in each condition, defined as the difference in accuracy between matched control trials and induction trials (cost = control ACC − induction ACC), where a positive value indicates that induction impaired performance relative to the item-matched baseline. This analysis was conducted on the 15 matched participant pairs for whom both induction and control trial accuracy were available for the same items within each condition, as determined by the counterbalancing structure of the design.

Under low semantic accessibility, induction costs were statistically significant for both weak induction (*M* = 0.122, *SD* = 0.137, 95% *CI* [0.046, 0.198]; *t* (14) = 3.460, *p* = .004, *d* = 0.893) and strong induction (*M* = 0.072, *SD* = 0.099, 95% *CI* [0.017, 0.127]; *t* (14) = 2.827, *p* = .013, *d* = 0.730), indicating that induction reliably impaired performance relative to item-matched baselines in this condition. Under high semantic accessibility, induction costs were non-significant for both weak induction (*M* = 0.072, *SD* = 0.160, 95% *CI* [−0.016, 0.161]; *t* (14) = 1.746, *p* = .103, *d* = 0.451) and strong induction (*M* = 0.067, *SD* = 0.148, 95% *CI* [−0.016, 0.149]; *t* (14) = 1.740, *p* = .104, *d* = 0.449), suggesting that the fixation effect was attenuated when semantic accessibility was high.

Direct between-condition comparisons of induction cost did not reach significance for either strong induction (*t* (14) = −0.133, *p* = .896, *d* = −0.034, 95% *CI* [−0.195, 0.172]) or weak induction (*t* (14) = −0.951, *p* = .358, *d* = −0.246, 95% *CI* [−0.163, 0.063]). This result likely reflects the limited statistical power of the matched-pair comparison (*n* = 15 pairs per condition), which is considerably smaller than the full within-subjects structure (*N* = 30) used in the primary ANOVA. Nonetheless, the pattern across all four cost estimates is directionally consistent with the attenuation hypothesis: induction costs were reliably greater than zero under low accessibility but not under high accessibility, and the point estimates were numerically smaller in the high-accessibility conditions (strong: *M* = 0.067 vs. 0.072; weak: *M* = 0.072 vs. 0.122). Taken together, the cost analysis provides descriptive support for the attenuation account, while the primary inferential basis for this conclusion remains the significant Accessibility × Induction Strength interaction in the full repeated-measures ANOVA (*F* (1, 29) = 4.88, *p* = .035, *η_p_*^2^ = 0.14), which uses the complete within-subjects design and thus carries substantially greater statistical power.

**Figure 3:**
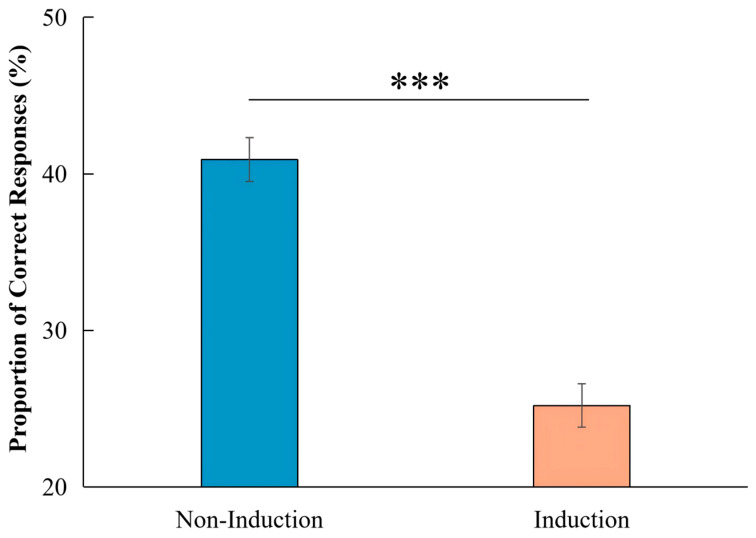
Proportion of correct responses in the test trial as a function of prior induction. Note. Error bars represent standard errors of the mean, *** *p* < .001.

To validate the induction manipulation, we compared performance between trials preceded by induction and those presented without induction (Figure 3). Participants solved significantly more items in the Non-Induction condition (*M* = 40.9%, *SE* = 2.1%) than in the Induction condition (*M* = 25.2%, *SE* = 1.4%; *t* (29) = 5.21, *p* < .001, *d* = 0.95, 95% *CI* [9.5%, 21.9%]). These results indicate that prior induction trials, when semantically overlapping with the test items, impaired creative performance rather than facilitating it.

## 4. Discussion

The present study examined how semantic accessibility and situational induction interact to shape creative problem solving. Our findings suggest that performance on a remote-association task may depend not on any single cognitive factor but on the joint influence of experience-driven heuristic patterns and the breadth of semantic accessibility. Critically, we observed the predicted interaction: strong induction impaired accuracy only when semantic accessibility was low, whereas high semantic accessibility largely eliminated induction costs. This pattern is consistent with the default–interventionist account of reasoning (Evans, 2006; Stanovich et al., 2004), which proposes that analytic intervention becomes possible only when the cognitive system has access to competing representations that challenge the initial heuristic response.

### 4.1. Semantic Accessibility and Attenuated Induction-Based Fixation

Under low semantic accessibility—where cue-solution associations are weak and semantic activation is narrow—participants were highly susceptible to experience-induced mental sets. Repeated reinforcement of a solution pattern strengthened reliance on the same heuristic approach, mirroring classical Einstellung findings where familiar strategies override problem-specific requirements (Luchins, 1942; Bilalić et al., 2010). Recent evidence further shows that insight-like problem solving requires the cognitive control system to suppress predominant but invalid ideas, and that failures of such inhibition contribute directly to fixation (Zhou et al., 2022). This is consistent with neurocognitive evidence showing that repeated strategy activation may reduce conflict-detection signals and promote cognitive inertia (Huang et al., 2019).

In contrast, under high semantic accessibility, induction strength no longer affected performance. High-accessibility items contain stronger associative links, providing richer access to semantic space. Prior work suggests that such rich semantic structure enhances associative diversity (Kenett, 2018; Kenett et al., 2016) and may support the suppression of misleading dominant associations (Luft et al., 2018). Within a heuristic–analytic framework, this richer representational landscape is consistent with the possibility that analytic processes are more likely to detect mismatches between the induced heuristic response and the task requirements, potentially enabling successful override. Thus, semantic accessibility may be associated with attenuated induction-based fixation, even when the heuristic response has been repeatedly reinforced. The induction cost analysis further corroborates this interpretation: fixation costs were reliably significant under low semantic accessibility but not under high semantic accessibility, a dissociation that persists even after controlling for item-level baseline difficulty. Although the present behavioral data are consistent with this interpretation, direct evidence for the underlying mechanisms—such as conflict detection or representational restructuring—awaits neurophysiological investigation.

It is important to note, however, that the present operationalization of semantic accessibility via associative frequency does not fully decouple semantic pathway breadth from overall item difficulty. Future research employing stimuli that vary in semantic network structure while holding baseline difficulty constant would be needed to isolate these contributions more cleanly. This account aligns with broader evidence from design creativity research, which shows that exposure to dominant solution structures can constrain exploratory search and increase the likelihood of fixation (Crilly & Cardoso, 2017).

### 4.2. Resolving Conflicting Findings on the Role of Semantic Knowledge

The observed interaction—whereby induction impaired performance under low semantic accessibility but not under high semantic accessibility—reconciles a long-standing inconsistency in the creativity literature. Semantic knowledge has been shown to impede creativity by reinforcing dominant associations that block access to novel solutions (Gupta et al., 2012; Duncker, 1945; Wiley, 1998). Neuroimaging evidence further shows that such conceptual fixation is reflected in stable neural activation patterns that hinder the generation of more flexible or remote associations (Frith et al., 2022). Yet other work demonstrates that rich semantic networks promote remote associations and originality (Benedek et al., 2012, 2013; Kenett, 2018). The present results clarify this paradox: semantic knowledge does not have a globally positive or negative effect but instead interacts with experiential context. When heuristic defaults are strong and semantic accessibility is low, knowledge amplifies fixation; when the semantic system provides broader access to alternative interpretations, knowledge enhances flexibility and facilitates insight. This explanation aligns with multi-source interference frameworks (Kershaw & Ohlsson, 2004), which posit that creative difficulty stems from the convergence of multiple interacting constraints.

### 4.3. Implications for Models of Insight and Creative Cognition

The present findings highlight the importance of considering semantic accessibility and associative availability as potential moderators of mental-set formation. Traditional insight theories emphasize constraint relaxation and representational change, but our results indicate that the likelihood of such restructuring may depend on the semantic landscape available during problem solving. Rich semantic networks offer the raw material for conflict detection and hypothesis revision, bridging the gap between associative accounts of creativity (Mednick, 1962; Kenett, 2018) and executive-control perspectives emphasizing inhibition and conflict monitoring (Luft et al., 2018; Morrison et al., 2017). This integrated explanation offers a theoretically plausible account of the possible pathways linking knowledge structure, experience, and creative flexibility, though causal claims about specific cognitive mechanisms remain to be tested directly in future work. At a practical level, these findings suggest that interventions aimed at broadening semantic accessibility—such as exposure to diverse associative contexts, vocabulary enrichment, or analogical training—may help individuals maintain cognitive flexibility even when prior experience creates strong heuristic defaults. In educational settings, this implies that supporting richer semantic networks may be as important as teaching explicit problem-solving strategies for fostering creative thinking. In professional domains prone to Einstellung effects, such as medical diagnosis or engineering design, the present results suggest that deliberately expanding the semantic and conceptual context available to practitioners could reduce experience-driven fixation and support more flexible problem solving.

## 5. Limitations and Future Directions

Although the sample was adequately powered to detect the predicted interaction, the relatively small sample size (*N* = 30) may limit the generalizability of these findings to broader populations. Future work with larger and more diverse samples would strengthen confidence in the robustness of the observed effects. Future work should explore individual differences in semantic network structure and cognitive flexibility that may modulate susceptibility to induction. The CRAT provides tight control over semantic relationships, but generalizing the observed interactions to other forms of creative problem solving—such as design fixation (Jansson & Smith, 1991; Purcell & Gero, 1996) or compound insight tasks (Sio & Ormerod, 2009; Morrison et al., 2017)—would strengthen the theoretical account. Neurophysiological measures could further clarify whether higher semantic accessibility facilitates conflict detection, eases inhibition of the induced heuristic, or accelerates representational change.

## 6. Conclusions

Together, the finding that semantic accessibility is associated with differential susceptibility to experience-induced heuristic fixation is consistent with the view that creative performance arises from the interaction of semantic knowledge and prior experience. Low semantic accessibility was associated with greater susceptibility to heuristic fixation, whereas high semantic accessibility was associated with attenuated fixation costs—a pattern consistent with semantic accessibility supporting analytic intervention and flexible problem solving. By identifying how semantic accessibility modulates the impact of situational induction, the present study is consistent with a unified account of how knowledge can both constrain and enable creativity, advancing theoretical models of insight and creative cognition.

## Figures and Tables

**Table 1:** Example stimulus sets for each experimental condition.

Conditions	Initial Induction	Enhance Induction 1	Enhance Induction 2	Enhance Induction 3	Test
LA	看、校、级 (高)	看、级、楼 (高)	看、楼、手 (高)	看、手、明 (高)	**查**看 (inspect, 9) **查**明 (clarify, 3) **查**阅 (consult, 5)
LW	年、习、期 (学)	年、期、识 (学)	-----	-----	**常**年 (all year round, 10) **常**识 (common sense, 7) **常**规 (routine, 8)
HA	立、作、举 (创)	立、新、举 (创)	立、举、始 (创)	立、始、设 (创)	**建**立 (establish, 336) **建**设 (construct, 598) **建**筑 (building, 172)
HW	头、军、此 (从)	头、此、来 (从)	-----	-----	**回**头 (turn back, 151) **回**来 (come back, 228) **回**答 (answer, 295)

## Data Availability

The datasets used and/or analyzed during the current study are available from the corresponding author on reasonable request.
